# Utility of the EULAR Sjögren syndrome disease activity index in Japanese children: a retrospective multicenter cohort study

**DOI:** 10.1186/s12969-020-00458-1

**Published:** 2020-09-17

**Authors:** Naomi Iwata, Minako Tomiita, Ichiro Kobayashi, Yusaburo Inoue, Yukiko Nonaka, Nami Okamoto, Hiroaki Umebayashi, Ryoki Hara, Yasuhiko Ito, Yasunori Sato, Masaaki Mori

**Affiliations:** 1 Department of Infection and Immunology, Aichi Children’s Health and Medical Center, Obu, Japan; 2 The Japan Pediatric Sjögren’s Syndrome Study Group, Tokyo, Japan; 3 Department of Pediatrics, Shimoshizu National Hospital, Yotsukaido, Japan; 4 Center for Pediatric Allergy and Rheumatology, KKR Sapporo Medical Center, Sapporo, Japan; 5 Department of Allergy and Rheumatology, Chiba Children’s Hospital, Chiba, Japan; 6 Department of Pediatrics, Kagoshima University Hospital, Kagoshima, Japan; 7 Department of Pediatrics, Osaka Medical College, Osaka, Japan; 8 Department of General Pediatrics, Miyagi Children’s Hospital, Sendai, Japan; 9 Department of Pediatrics, Yokohama City University School of Medicine, Yokohama, Japan; 10 Department of Pediatrics, Nippon Medical School, Tokyo, Japan; 11 Department of Preventive Medicine and Public Health, Keio University School of Medicine, Tokyo, Japan; 12 Department of Lifetime Clinical Immunology, Graduate School of Medical and Dental Sciences, Tokyo Medical and Dental University, Tokyo, Japan

**Keywords:** Primary Sjögren’s syndrome, Children, ESSDAI, Treatment

## Abstract

**Background:**

The European League Against Rheumatism (EULAR) Sjögren Syndrome Disease Activity Index (ESSDAI) has been utilized to assess Sjögren syndrome-related systemic involvement in adult patients. To date, however, the ESSDAI has not been validated in children with primary Sjögren’s syndrome. This study evaluated the applicability of the ESSDAI to Japanese children with primary Sjögren’s syndrome.

**Methods:**

The medical records of children who had been diagnosed with Sjogren syndrome at age ≤ 16 years between June 2011 and October 2016 were collected, and their ESSDAIs at initial presentation were calculated. Clinical symptoms and treatment regimens were surveyed by questionnaire, and patients were divided into groups based on ESSDAI and glucocorticoid dosages. The associations of ESSDAI scores with treatment regimens were analyzed statistically.

**Results:**

The study subjects included 31 children (3 boys, 28 girls) with primary Sjögren’s syndrome. Their median age at disease onset was 10 years (interquartile range [IQR], 8–13 years), and their median initial ESSDAI was 7.0 (IQR; 5.0–15.0). ESSDAI-determined disease activity was high in nine patients (29.0%), moderate in 15 (48.4%), and low in seven (22.6%). During the first year after their initial visit, 14 patients (45.2%) were treated with prednisolone (PSL) and six (19.4%) with immunosuppressants. Dose of PSL was significantly associated with ESSDAI score. Median ESSDAI score was significantly higher in patients treated with high/medium- than with no/low-dose PSL (16.5 [IQR 10.5–18.0] vs 5.0 [IQR 3.0–8.5]). Eight (66.7%) of 12 patients administered medium/high-dose PSL and one (5.3%) of 19 administered no/low-dose PSL had high disease activity on ESSDAI.

**Conclusion:**

Disease activity assessed by ESSDAI tended to be consistent with disease activity assessed by pediatric rheumatologists in determining treatment regimens. ESSDAI is useful for assessing disease activity in Japanese children with primary Sjögren’s syndrome.

## Background

Sjögren’s syndrome is an autoimmune exocrinopathy primarily affecting the salivary and lachrymal glands. Sjögren’s syndrome has been classified into two subsets: primary SS (pSS), which develops alone, and secondary SS, which develops in association with other connective tissue diseases such as systemic lupus erythematosus (SLE) and rheumatoid arthritis ●[1]. The pathological characteristics and autoantibody profiles of childhood and adult pSS are similar ●[2], suggesting that childhood pSS represents an early-onset counterpart of adult pSS or an early stage of pSS. Most pediatric patients with pSS, however, lack sicca symptoms, and are diagnosed on the basis of systemic symptoms and/or recurrent parotitis associated with pSS ●[3–6].

The SS Task Force Group steering committee of the European League Against Rheumatism (EULAR) has formulated a Sjögren Syndrome Disease Activity Index (ESSDAI) to assess SS-related systemic involvement [7] (Supplementary Table 1). The ESSDAI has been found to correlate with other clinical indicators of pSS, including physician’s global assessment, fever and lymphadenopathy [8, 9]. In addition, the items of the ESSDAI have been found useful for the diagnosis of pSS in adults [10] and are included in the American College of Rheumatology (ACR)/EULAR classification criteria for patients with pSS. To date, however, the ESSDAI has not been validated in children with pSS [11, 12].

Moreover, because therapeutic regimens have not been standardized in pSS patients including pediatric patients, they are based on expert opinions and the experience of individual attending physicians [13]. Accordingly, the selected treatment reflects any complications and disease activity in each patient.

## Methods

### Aim

The primary aim of this study was to evaluate the usefulness of the ESSDAI in children with pSS. The ESSDAI at initial visit was determined for each patient, and the associations between ESSDAI scores and treatment regimens were analyzed retrospectively.

### Study setting & patients

This was a retrospective multicenter cohort study involving the Japan Pediatric Sjögren’s Syndrome Study Group (JPSSG), consisting of nine pediatric rheumatologists organized as a subcommittee of a Scientific Research Group for Pediatric Rheumatic Diseases and with the support of the Japanese Ministry of Health, Labour and Welfare.

The medical records of children who had been diagnosed with SS at age ≤ 16 years between June 2011 and October 2016 were collected from nine medical institutions belonging to the JPSSG. These medical records were retrospectively reviewed by the members of the JPSSG at a face-to-face consensus expert meeting. Patients that were unanimously diagnosed by multiple physicians as pSS and patients diagnosed by multiple physicians of pSS but not unanimously, thus were diagnosed as suspected pSS, were included in the present study. The main characteristics for diagnosis of patients included in this study are shown in Supplementary Table 2. Patients who first visited these institutions ≥5 years after disease onset and those who developed other rheumatic diseases within 6-months after the diagnosis of pSS were excluded.

### Study design

#### Data collection

Data of children with pSS who fulfilled the above inclusion criteria were retrospectively collected from responses to questionnaires administered to the members of the JPSSG. These questionnaires assessed the baseline characteristics, clinical and laboratory measurements including ESSDAI domains, and treatment during the first 12 months after initial presentation of all included patients. Disease onset was defined as the emergence of the first symptoms presumably associated with pSS. Clinical symptoms observed at the first visit to a pediatric rheumatologist were divided into glandular symptoms, such as sicca symptoms, and extraglandular symptoms. ESSDAI at initial presentation was retrospectively calculated from medical records by each JPSSG member. Initial ESSDAI was defined as the maximum score during the 6 months after the first visit. Disease activities were graded as high (ESSDAI ≥14), moderate (5–13) and low (≤4) [7].

#### Relationship between ESSDAI and treatment

To evaluate the relationship between ESSDAI score and treatment, glucocorticoid dosage and treatment with immunosuppressants within 1 year after the initial visit were compared among the groups of patients with high, medium, and mild disease activity, as determined by ESSDAI. Glucocorticoid dosages were graded as high (prednisolone [PSL] ≥ 0.5 mg/kg/day or equivalent), medium (PSL ≥0.2 but < 0.5 mg/kg/day or equivalent) and low (PSL < 0.2 mg/kg/day or equivalent). Median ESSDAI score and the proportion of patients with high disease activity were compared between the groups of patients receiving high/medium-dose PSL and no/low-dose PSL.

To assess ESSDAI-associated factors influencing treatment decisions, the number of patients scored as active in each ESSDAI domain was compared between the groups of patients receiving high/medium and no/low dose PSL. In addition, the numbers of ESSDAI domains scored as active and the maximum level of activity achieved by each patient in any of the 12 ESSDAI domains were compared in the high/medium-dose and no/low-dose PSL groups.

#### Effect of sicca symptoms

The relationships of sicca symptoms with total ESSDAI scores and treatment regimens were analyzed by comparing these factors in patients with and without sicca symptoms.

#### Statistical analysis

Demographic characteristics and ESSDAI scores were reported as medians and first and third quartiles. Categorical variables in the two groups were compared using Fisher’s exact tests, and continuous variables were compared using Mann-Whitney U tests. All comparisons were planned and the tests were two-tailed, with *p*-values < 0.05 considered statistically significant. All statistical analyses were performed using the EZR software package (Saitama Medical Center, Jichi Medical University, Saitama, Japan) [14].

## Results

### Clinical characteristics

Of the 37 patients diagnosed with pSS during the study period, six were excluded, two because their first visits were delayed ≥5 years after disease onset, and four because of development of other rheumatic diseases within 6-months after the first visit, including three who developed systemic lupus erythematosus (SLE) and one who developed rheumatoid factor (RF)-positive juvenile idiopathic arthritis. The remaining 31 patients (3 boys and 28 girls) were enrolled in the present study. Their median age at diagnosis was 10 years (interquartile range [IQR] 8–13 years), and the median interval between disease onset and the first visit was 4.0 months (IQR, 1.0–9.8 months).

### Clinical symptoms at first visit

Before their first visit to a pediatric rheumatologist, 15 patients (48.4%) had glandular symptoms (Table 1), including nine (29.0%) each with sicca symptoms and recurrent parotitis. Twenty-nine (93.5%) patients had extraglandular symptoms, with fever being the most frequent, being observed in 19 (61.3%) patients followed by arthritis/arthralgia (45.2%), malaise, lymphadenopathy, rash, and Raynaud’s phenomenon.

**Table 1:** Clinical symptoms and ESSDAI items of sicca-positive and -negative patients at baseline

	Clinical symptoms at first visit			Initial ESSDAI
	All patients (*n* = 31)	Sicca (+) (*n* = 9)	Sicca (−) (*n* = 22)	(12 domains)
Glandular involvement	15 (48.4%)			
Sicca symptoms	9 (29.0%)	9	0	
Recurrent parotitis (glandular)	9 (29.0%)	3	6	10 (32.3%)
Extraglandular symptoms	29 (93.5%)	9	20	
Constitutional (fever)	19 (61.3%)	5	14	18 (58.1%)
Lymphadenopathy	7 (22.6%)	1	6	9 (29.0%)
Arthritis/arthralgia	14 (45.2%)	4	10	9 (29.0%)
Cutaneous (annular erythema)	7 (22.6%)	3	4	9 (29.0%)
Cutaneous (purpura)	6 (19.4%)	4	2	
Peripheral neurologic symptoms	1 (3.2%)	1	0	2 (6.5%)
Central nervous system symptoms	1 (3.2%)	1	0	3 (9.7%)
Raynaud’s symptoms	5 (16.1%)	2	3	
General fatigue	13 (41.9%)	3	10	
Symptoms/data of other ESSDAI domains				
Pulmonary	0 (0%)	0	0	0 (0%)
Renal	0 (0%)	0	0	1 (3.2%)
Muscle	0 (0%)	0	0	0 (0%)
Hematological				3 (9.7%)
Biological				18 (58.1%)

The last column, Initial ESSDAI, lists number (%) of patients who were positive for each disease feature on the Initial ESSDAI within 6 months of the first visit

Abbreviations: *ESSDAI* EULAR Sjögren Syndrome Disease Activity Index, *EULAR* European League Against Rheumatism

### Initial ESSDAI

The overall median initial ESSDAI was 7.0 (IQR; 5.0–15.0). Disease activity, as determined by ESSDAI, was high (≥14) in nine (29.0%) patients, medium (5–13) in 15 (48.4%) and low (≤4) in seven (22.6%). The proportions of boys, age at diagnosis, and interval between disease onset and the first visit did not differ significantly among these three disease activity groups.

The most frequently involved ESSDAI domains were constitutional and biological domains in 18 patients (58.1%), followed by glandular in 10 (32.3%), articular (arthritis and arthralgia) in nine (29%) and cutaneous in nine (29%). Other involved domains included renal in one patient (3.2%), the peripheral nervous system in two (6.5%), and the central nervous system and hematological in three each (9.7%) (Table 1).

### Medication during the first year

Fourteen patients (45.2%) were treated with glucocorticoids during the first year after their initial visit; including four patients with high-dose, eight with medium-dose, and two with low-dose PSL, whereas the other 17 patients did not receive PSL. During the same period, six patients (19.4%) received immunosuppressants, including two who both received mizoribine, two treated with methotrexate, one treated with mycophenolate mofetil and one treated with tacrolimus.

### Relationship between ESSDAI and treatment

**Fig. 1:**
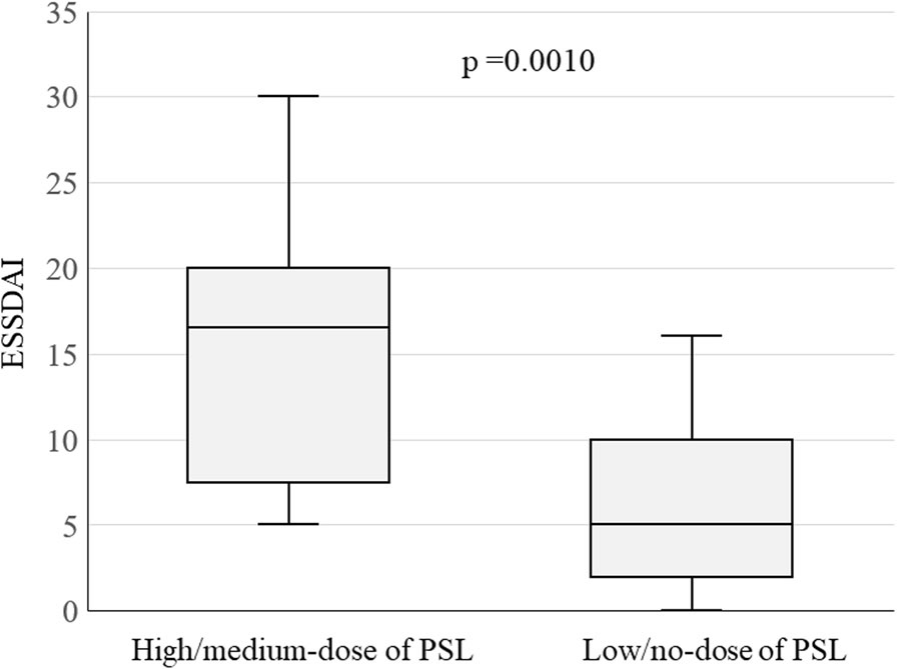
Comparison of ESSDAI scores in children with pSS treated with high/medium-dose and no/low-dose PSL. ESSDAI was significantly higher in the former than in the latter group (*p* = 0.0010)

Of the nine patients with high disease activity at initial presentation, eight (89%) were treated with PSL, including three with high-dose and five with medium-dose PSL. Of the 15 patients with moderate disease activity, six (40%) were treated with PSL; including one with high dose, three with medium dose, and two with low dose PSL. None of the patients with low disease activity was treated with PSL. The proportions of patients treated with PSL differed significantly by disease activity group on ESSDAI (*p* = 0.0010). Median ESSDAI score was significantly higher in patients treated with high/medium-dose than with no/low-dose PSL (16.5 [IQR 10.5–18.0] vs 5.0 [IQR 3.0–8.5], *p =* 0.0010) (Fig. 1). In addition, the proportion of patients with high disease activity (≥14) was significantly higher in the group receiving high or moderate dose PSL than in the group receiving no or low dose PSL (66.7% vs 5.3%, *P* = 0.00047). Immunosuppressants were also administered to four (44%) of nine patients with high disease activity score, and two (13%) of the 15 with moderate activity score, but none of the patients with low disease activity score.

Table 2 shows the numbers of patients scored as active and those with moderate or high activity in each domain, and those in high/moderate and low/no PSL dose group. All patients with visceral involvement (renal, peripheral nervous system, and central nervous system) and all with moderate or high activity in the lymphadenopathy, glandular, and articular domains were treated with high/medium-dose PSL. Patients active in three or more domains and those with high activity in any ESSDAI domain were more likely to receive high/medium-dose than low/no-dose PSL (Table 3).

**Table 2:** Numbers of patients positive on each ESSDAI domain and positive for moderate/high activity on each domain as a function of level of treatment with PSL

	Patients with positive activity level			Patients with moderate/high activity level	
	All patients (*n* = 31)	High/medium dose PSL (*n* = 12)	No/low dose PSL (*n* = 19)	High/medium dose PSL (*n* = 12)	No/low dose PSL (*n* = 12)
**ESSDAI domain**					
Constitutional	18	9 (50.0%)	9 (50.0%)	5	4
Lymphadenopathy	9	3 (33.3%)	6 (66.7%)	1	0
Glandular	10	5 (50.0%)	5 (50.0%)	4	0
Articular	9	4 (44.4%)	5 (55.6%)	1	0
Cutaneous	9	6 (66.7%)	3 (33.3%)	2	2
Pulmonary	0	0	0	0	0
Renal	1	1 (100%)	0	0	0
Muscular	0	0	0	0	0
PNS	2	2 (100%)	0	1	0
CNS	3	3 (100%)	0	3	0
Hematological	3	2 (66.7%)	1 (33.3%)	2	1
Biological	18	10 (55.6%)	8 (44.4%)	9	7

Abbreviations: *ESSDAI* EULAR Sjögren Syndrome Disease Activity Index, *EULAR* European League Against Rheumatism, *PNS* peripheral nervous system, *CNS* central nervous system, *PSL* prednisolone

Glucocorticoid dosages were graded as high (prednisolone (PSL) ≥ 0.5 mg/kg/day or equivalent), medium (PSL ≥0.2 but < 0.5 mg/kg/day or equivalent) and low (PSL < 0.2 mg/kg/day or equivalent)

**Table 3:** Relationship between ESSDAI domain and PSL dosage

	All (*n* = 31)	High/medium dose PSL group (*n* = 12)	No/low dose PSL group (*n* = 19)
Number of ESSDAI domains scored as active			
0	1	0 (0%)	1 (100%)
1	7	0 (0%)	7 (100%)
2	10	3 (30.0%)	7 (70.0%)
≥ 3	13	9 (69.2%)	4 (30.8%)
Maximum level of activity achieved in any of the 12 ESSDAI domains			
No	1	0 (0%)	1 (100%)
Low	8	0 (0%)	8 (100%)
Moderate	19	9 (47.3%)	10 (52.6%)
High	3	3 (100%)	0 (0%)

Abbreviations: *ESSDAI* EULAR Sjögren Syndrome Disease Activity Index, *EULAR* European League Against Rheumatism, *PSL* prednisolone

Glucocorticoid dosages were graded as high (prednisolone (PSL) ≥ 0.5 mg/kg/day or equivalent), medium (PSL ≥0.2 but < 0.5 mg/kg/day or equivalent) and low (PSL < 0.2 mg/kg/day or equivalent)

One patient did not have any active domains

### Comparison of ESSDAI and treatment in patients with and without sicca symptoms

Sicca symptoms were observed in nine (29.0%) of the 31 patients at their first visit. The extraglandular symptoms observed in patients with and without sicca symptoms are shown in Table 1. ESSDAI did not differ significantly in patients with (7.0 [IQR 2.0–16.0]) and without (6.5 [IQR 5.0–13.8]) sicca symptoms (*p* = 0.585), nor did treatment with PSL or immunosuppressants.

## Discussion

Although several classification criteria for pSS have been published, none to date has been validated in children. Thus, we included patients diagnosed as SS by consensus of expert pediatric rheumatologists. The present study found that a high proportion of patients with childhood pSS had constitutional symptoms on the ESSDAI, including fever (61.3%), whereas only 29% had sicca symptoms at presentation. Other extraglandular symptoms in our patient cohort included arthralgia (45.2%), recurrent parotitis (29.0%), lymphadenopathy (22.6%) and annular erythema (22.6%). These findings were similar to previous reports about children with pSS ●[3–6]. In contrast, over 90% of adults who present with pSS show sicca symptoms, although they less frequently show systemic or extraglandular symptoms, especially constitutional symptoms, lymphadenopathy, and cutaneous symptoms on the ESSDAI [8, 15] (Supplementary Table 3). Almost 70% of patients in our study had multiple active domains or developed more than moderate activity in any individual domain. As a result, a higher proportion (77.4%) of children than adults with pSS presented with moderate or high disease activity (ESSDAI ≥5) [9] (Supplementary Table 4), in agreement with a previous report from a single institution [12]. Of note, our results suggested that sicca symptoms were not associated with extraglandular symptoms and ESSDAI in children with pSS.

In addition to assessing disease activity in daily clinical practice, the ESSDAI is also used to evaluate the effects of treatment, including biologic therapy, in patients enrolled in clinical trials [16]. To date, few randomized controlled trials have evaluated the effects of treatment on extraglandular symptoms in patients with pSS. Thus, the decision to treat with glucocorticoids and/or immunosuppressants is based on expert opinions and/or the experience of individual attending physicians [13, 17]. The present study demonstrated that the use of glucocorticoids and/or immunosuppressants correlated with ESSDAI scores. Because this survey was conducted at multiple facilities, possible bias in treatment choice among physicians was minimized. In addition, ESSDAI score itself did not influence the choice of treatment by each attending physician, as the treatment was decided prior to assessment of ESSDAI. Nevertheless, all patients with moderate or high activity in the lymphadenopathy, glandular, articular, peripheral nervous system and central nervous system domains of the ESSDAI were treated with high/medium doses of PSL. In addition, patients scored as active in three or more domains and those with higher activity in any domain tended to be treated with high/medium dose PSL. These findings suggest that the domains of ESSDAI are appropriate for the assessment of overall SS disease activity in children. In contrast, treatment with glucocorticoids and/or immunosuppressants did not differ between patients with and without sicca symptoms, indicating that the choice of treatment reflected the activity of systemic or extraglandular disease rather than the presence of sicca symptoms.

The present study had several limitations, including its retrospective design and small sample size. Moreover, this study could not assess changes in ESSDAI after treatment. It would be desirable to monitor these patients longitudinally to track and examine any changes over time in ESSDAI score and treatment in pediatric patients with pSS. Another limitation was that patients with muscle and pulmonary lesions were not included, because these lesions are quite rare in children [3–6].

In conclusion, ESSDAI score correlates with the strength of treatment, suggesting that ESSDAI score reflects the disease activity in individual children with pSS. ESSDAI may be useful in determining a treatment strategy for these patients in daily clinical practice. A formal international study is needed to validate the ESSDAI in children diagnosed with primary Sjogren Syndrome.

## Supplementary information

**Additional file 1: Table S1.** The EULAR Sjögren’s Syndrome Disease Activity Index (ESSDAI): Domain and item definitions and weights. **Table S2.** Diagnosis of Sjogren syndrome and main clinical characteristics during the course for diagnosis

**Additional file 2: Table S3.** Percentage of patients scored as active at diagnosis in the individual clinical ESSDAI domains: Comparison with adult patients reported previously. **Table S4.** Percentage of patients in each ESSDAI score categories and level of ESSDAI activity: Comparison with adult patients reported previously.

**Additional file 3: Supplementary Table.** The type of immunosuppressants are listed below and shown in the manuscript (Page 11, line 1-4).

## Data Availability

Although the data used in this manuscript are available from the corresponding author on reasonable request, these are not publicly available for ethical and privacy reasons.

## Abbreviations

ACR: American College of Rheumatology
EULAR: European League Against Rheumatism
ESSDAI: EULAR Sjögren Syndrome Disease Activity Index
PSL: Prednisolone
IQR: Interquartile range
SLE: Systemic lupus erythematosus
JPSSG: The Japan Pediatric Sjögren’s Syndrome Study Group
