# Supplementary material for: Utility of the EULAR Sjögren syndrome disease activity index in Japanese children: a retrospective multicenter cohort study
Source: Pediatr Rheumatol Online J. 2020 Sep 17;18:73. doi: 10.1186/s12969-020-00458-1 (PMC7499954; doi:10.1186/s12969-020-00458-1)
Supplement: Supplementary file 1 — Additional file 1: Table S1. The EULAR Sjögren’s Syndrome Disease Activity Index (ESSDAI): Domain and item definitions and weights. Table S2. Diagnosis of Sjogren syndrome and main clinical characteristics during the course for diagnosis [file 12969_2020_458_MOESM1_ESM.docx]

Supplemental Table 1: The EULAR Sjögren’s Syndrome Disease Activity Index (ESSDAI): Domain and item definitions and weights [1].

| **Domain [Weight]** | **Activity level** | **Description** |
| --- | --- | --- |
| **Constitutional** [3]  *Exclusion of fever of infectious origin and voluntary weight loss* | No = 0 | Absence of the following symptoms |
|  | Low = 1 | Mild or intermittent fever (37.5°–38.5°C)/night sweats and/or involuntary weight loss of 5 to 10% of body weight |
|  | Moderate = 2 | Severe fever (>38.5°C)/night sweats and/or involuntary weight loss of >10% of body weight |
| **Lymphadenopathy** [4]  *Exclusion of infection* | No = 0 | Absence of the following features |
|  | Low = 1 | Lymphadenopathy ≥ 1 cm in any nodal region or ≥ 2 cm in inguinal region |
|  | Moderate = 2 | Lymphadenopathy ≥ 2 cm in any nodal region or ≥ 3 cm in inguinal region, and/or splenomegaly (clinically palpable or assessed by imaging) |
|  | High = 3 | Current malignant B-cell proliferative disorder |
| **Glandular** [2]  *Exclusion of stone or infection* | No = 0 | Absence of glandular swelling |
|  | Low = 1 | Small glandular swelling with enlarged parotid (≤ 3 cm), or limited submandibular or lachrymal swelling |
|  | Moderate = 2 | Major glandular swelling with enlarged parotid (> 3 cm), or important submandibular or lachrymal swelling |
| **Articular** [2]  *Exclusion of osteoarthritis* | No = 0 | Absence of currently active articular involvement |
|  | Low = 1 | Arthralgias in hands, wrists, ankles and feet accompanied by morning stiffness (>30 min) |
|  | Moderate = 2 | 1 to 5 (of 28 total count) synovitis |
|  | High = 3 | ≥ 6 (of 28 total count) synovitis |
| **Cutaneous** [3]  *Rate as “No activity” stable long-lasting features related to damage* | No = 0 | Absence of currently active cutaneous involvement |
|  | Low = 1 | Erythema multiforma |
|  | Moderate = 2 | Limited cutaneous vasculitis, including urticarial vasculitis, or purpura limited to feet and ankle, or subacute cutaneous lupus |
|  | High = 3 | Diffuse cutaneous vasculitis, including urticarial vasculitis, or diffuse purpura, or ulcers related to vasculitis |
| **Pulmonary** [5]  *Rate as “No activity” stable long-lasting features related to damage, or respiratory involvement not related to the disease (tobacco use etc.)* | No = 0 | Absence of currently active pulmonary involvement |
|  | Low = 1 | Persistent cough or bronchial involvement with no radiographic abnormalities on radiography  Or radiological or HRCT evidence of interstitial lung disease with: No breathlessness and normal lung function test. |
|  | Moderate = 2 | Moderately active pulmonary involvement, such as interstitial lung disease shown by HRCT with shortness of breath on exercise (NHYA II) or abnormal lung function tests restricted to: 70% >DLCO≥ 40% or 80%>FVC≥60% |
|  | High = 3 | Highly active pulmonary involvement, such as interstitial lung disease shown by HRCT with shortness of breath at rest (NHYA III, IV) or with abnormal lung function tests: DLCO< 40% or FVC< 60% |
| **Renal** [5]  *Rate as “No activity” stable long-lasting features related to damage, and renal involvement not related to the disease.*  *If biopsy has been performed, please rate activity based on histological features first* | No = 0 | Absence of currently active renal involvement with proteinuria< 0.5 g/d, no hematuria, no leukocyturia, no acidosis, or long-lasting stable proteinuria due to damage |
|  | Low = 1 | Evidence of mild active renal involvement, limited to tubular acidosis without renal failure or glomerular involvement with proteinuria (between 0.5 and 1 g/d) and without hematuria or renal failure (GFR ≥60 ml/min) |
|  | Moderate = 2 | Moderately active renal involvement, such as tubular acidosis with renal failure (GFR <60 ml/min) or glomerular involvement with proteinuria between 1 and 1.5 g/d and without hematuria or renal failure (GFR ≥60 ml/min) or histological evidence of extra-membranous glomerulonephritis or important interstitial lymphoid infiltrate |
|  | High = 3 | Moderately active renal involvement, such as tubular acidosis with renal failure (GFR <60 ml/min) or glomerular involvement with proteinuria between 1 and 1.5 g/d and without hematuria or renal failure (GFR ≥60 ml/min) or histological evidence of extra-membranous glomerulonephritis or important interstitial lymphoid infiltrate |
| **Muscular** [6]  *Exclusion of weakness due to corticosteroids* | No = 0 | Absence of currently active muscular involvement |
|  | Low = 1 | Mild active myositis shown by abnormal EMG or biopsy with no weakness and creatine kinase (N <CK ≤ 2N) |
|  | Moderate = 2 | Moderately active myositis proven by abnormal EMG or biopsy with weakness (maximal deficit of 4/5), or elevated creatine kinase (2N<CK ≤4N), |
|  | High = 3 | Highly active myositis shown by abnormal EMG or biopsy with weakness (deficit ≤ 3/5) or elevated creatine kinase (>4N) |
| **PNS** [5]  *Rate as “No activity” stable long-lasting features related to damage or PNS involvement not related to the disease* | No = 0 | Absence of currently active PNS involvement |
|  | Low = 1 | Mild active peripheral nervous system involvement, such as pure sensory axonal polyneuropathy shown by NCS or trigeminal (V) neuralgia |
|  | Moderate = 2 | Moderately active peripheral nervous system involvement shown by NCS, such as axonal sensory-motor neuropathy with maximal motor deficit of 4/5, pure sensory neuropathy with presence of cryoglobulinemic vasculitis, ganglionopathy with symptoms restricted to mild/moderate ataxia, inflammatory demyelinating polyneuropathy (CIDP) with mild functional impairment (maximal motor deficit of 4/5or mild ataxia),  Or cranial nerve involvement of peripheral origin (except trigeminal (V) neuralgia) |
|  | High = 3 | Highly active PNS involvement shown by NCS, such as axonal sensory-motor neuropathy with motor deficit ≤3/5, peripheral nerve involvement due to vasculitis (mononeuritis multiplex etc.), severe ataxia due to ganglionopathy, inflammatory demyelinating polyneuropathy (CIDP) with severe functional impairment: motor deficit ≤3/5 or severe ataxia |
| **CNS** [5]  *Rate as “No activity” stable long-lasting features related to damage or CNS involvement not related to the disease* | No = 0 | Absence of currently active CNS involvement |
|  | Low = 1 | Moderately active CNS features, such as cranial nerve involvement of central origin, optic neuritis or multiple sclerosis-like syndrome with symptoms restricted to pure sensory impairment or proven cognitive impairment |
|  | High = 3 | Highly active CNS features, such as cerebral vasculitis with cerebrovascular accident or transient ischemic attack, seizures, transverse myelitis, lymphocytic meningitis, multiple sclerosis-like syndrome with motor deficit. |
| **Hematological** [2]  *For anemia, neutropenia, and thrombopenia, only auto-immune cytopenia must be considered*  *Exclusion of vitamin or iron deficiency, drug-induced cytopenia* | No = 0 | Absence of auto-immune cytopenia |
|  | Low = 1 | Cytopenia of auto-immune origin with neutropenia (1000 < neutrophils < 1500/mm3), and/or anemia (10 < hemoglobin < 12 g/dl), and/or thrombocytopenia (100,000 < platelets < 150,000/mm3)  Or lymphopenia (500 < lymphocytes < 1000/mm3) |
|  | Moderate = 2 | Cytopenia of auto-immune origin with neutropenia (500 ≤ neutrophils ≤ 1000/mm3), and/or anemia (8 ≤ hemoglobin ≤ 10 g/dl), and/or thrombocytopenia (50,000 ≤ platelets ≤ 100,000/mm3)  Or lymphopenia (≤500/mm3) |
|  | High = 3 | Cytopenia of auto-immune origin with neutropenia (neutrophils < 500/mm3), and/or or anemia (hemoglobin < 8 g/dl) and/or thrombocytopenia (platelets <50,000/mm3) |
| **Biological** [1] | No = 0 | Absence of any of the following biological feature |
|  | Low = 1 | Clonal component and/or hypocomplementemia (low C4 or C3 or CH50) and/or hypergammaglobulinemia or high IgG level between 16 and 20 g/L |
|  | Moderate = 2 | Presence of cryoglobulinemia and/or hypergammaglobulinemia or high IgG level > 20 g/L, and/or recent onset hypogammaglobulinemia or recent decrease of IgG level (<5 g/L) |

CIDP= chronic inflammatory demyelinating polyneuropathy; CK= creatine kinase; CNS= central nervous system; DLCO= diffusing CO capacity;

EMG= electromyogram; FVC= forced vital capacity; GFR= glomerular filtration rate; Hb= hemoglobin; HRCT= high-resolution computed

tomography; IgG= immunoglobulin G; NCS= nerve conduction studies; NHYA= New York heart association classification; Plt= platelet;

PNS=peripheral nervous system;

Supplementary Table 2: Diagnosis of Sjogren syndrome and main clinical characteristics during the course for diagnosis

| No. of Patient | Diagnosis of SS by JPSSG members | Biopsy of Labial salivary gland | Autoantibody | | | | Symptoms and results of sicca at the investigation | Initial ESSDAI |
| --- | --- | --- | --- | --- | --- | --- | --- | --- |
|  |  |  | SS-A/Ro | SS-B/La | ANA | RF |  |  |
| 1 | Definitive | LS | **+** | **+** | **+** | **+** | + | 30 |
| 2 | Definitive | LS | **+** | **+** | **+** | **+** | + | 14 |
| 3 | Definitive | LI | **+** | **+** | **+** | **+** | - | 5 |
| 4 | Definitive | LS | **+** | **+** | **+** | **+** | - | 17 |
| 5 | Definitive | n.t. | **+** | **-** | **+** | **+** | - | 5 |
| 6 | Suspected | n.t. | **+** | **+** | **+** | **+** | + | 7 |
| 7 | Definitive | LS | **+** | **-** | **+** | **-** | + | 6 |
| 8 | Definitive | LS | **+** | **+** | **+** | **+** | + | 2 |
| 9 | Definitive | LS | **+** | **+** | **+** | **+** | - | 11 |
| 10 | Definitive | LS | **+** | **+** | **+** | **+** | - | 17 |
| 11 | Definitive | LS | **+** | **+** | **+** | **+** | - | 4 |
| 12 | Definitive | LS | **+** | **-** | **+** | **+** | + | 5 |
| 13 | Definitive | LS | **+** | **-** | **+** | **+** | - | 5 |
| 14 | Definitive | LS | **+** | **-** | **+** | **+** | + | 6 |
| 15 | Definitive | n.t. | **+** | **-** | **+** | **+** | - | 2 |
| 16 | Suspected | LS | **-** | **-** | **-** | **+** | + | 16 |
| 17 | Definitive | LS | **+** | **+** | **+** | + | - | 5 |
| 18 | Definitive | LI | **-** | **-** | **-** | **+** | + | 21 |
| 19 | Definitive | LI | **+** | **+** | **+** | **-** | + | 7 |
| 20 | Definitive | LI | **+** | **+** | **+** | **+** | - | 17 |
| 21 | Definitive | LI | **+** | **+** | **+** | **+** | + | 12 |
| 22 | Definitive | LS | **+** | **-** | **+** | - | - | 29 |
| 23 | Definitive | LS | **+** | **+** | **+** | **+** | + | 16 |
| 24 | Definitive | n.t. | **+** | **+** | **+** | - | + | 13 |
| 25 | Suspected | n.t. | **+** | **-** | **-** | **-** | + | 10 |
| 26 | Suspected | n.t. | **-** | **-** | **+** | **+** | + | 2 |
| 27 | Suspected | n.t. | **-** | **-** | **+** | **+** | + | 4 |
| 28 | Suspected | n.t. | **-** | **-** | **-** | **+** | - | 2 |
| 29 | Definitive | n.t. | **+** | **+** | **+** | **+** | + | 0 |
| 30 | Definitive | LS | **+** | **-** | **+** | **+** | - | 6 |
| 31 | Definitive | LI | **+** | **-** | **+** | **+** | - | 12 |

Abbreviations: SS, Sjögren's syndrome; JPSSG, Japan Pediatric Sjögren's Syndrome Study Group; ESSDAI, EULAR Sjögren syndrome disease activity index; ANA, antinuclear antibody; RF, rheumatoid factor; LS, lymphocytic sialadenitis; LI, lymphocyte infiltration; n.t., not tested

The second column lists the diagnosis of SS by JPSSG members. Patients voted as pSS by all specialist were diagnosed as "Definitive". Patients who were voted as pSS by multiple physicians but not by all physicians were diagnosed as "Suspected".

LS, lymphocytic sialadenitis, was defined as the sialadenitis with focus score of >1 foci/4mm^2^ in labial salivary gland biopsy.

LI, lymphocyte infiltration, was defined as infiltrations around the salivary duct with focus score of less than 1 focus/4mm^2^.

ANA more than a dilution of 1:160 or equal was defined as positive.

The last column, Initial ESSDAI, lists number (%) of patients who were positive for each disease feature on the Initial ESSDAI within 6 months of the first visit.
