# Supplementary material for: Utility of the EULAR Sjögren syndrome disease activity index in Japanese children: a retrospective multicenter cohort study
Source: Pediatr Rheumatol Online J. 2020 Sep 17;18:73. doi: 10.1186/s12969-020-00458-1 (PMC7499954; doi:10.1186/s12969-020-00458-1)
Supplement: Supplementary file 2 — Additional file 2: Table S3. Percentage of patients scored as active at diagnosis in the individual clinical ESSDAI domains: Comparison with adult patients reported previously. Table S4. Percentage of patients in each ESSDAI score categories and level of ESSDAI activity: Comparison with adult patients reported previously. [file 12969_2020_458_MOESM2_ESM.docx]

Supplementary Table 3:

Percentage of patients scored as active at diagnosis in the individual clinical ESSDAI domains: Comparison with adult patients reported previously [2]

| Individual ESSDAI domain  and degree of activity | Patients with activity at diagnosis (%) | | *P*-value* |
| --- | --- | --- | --- |
|  | Children  n = 31 | Adults [2]  n = 921 |  |
| Constitutional |  |  | 1.14e-13 |
| No | 13 (41.9) | 813 (88.3) |  |
| Any | 18 (58.1) | 78 (8.5) |  |
| Low | 9 (29) | 64 (7) |  |
| Moderate | 9 (29) | 14 (1.5) |  |
| Lymphadenopathy |  |  | 4.89e-06 |
| No | 22 (71) | 861 (93.5) |  |
| Any | 9 (29) | 60 (6.5) |  |
| Low | 8 (25.8) | 34 (3.7) |  |
| Moderate | 1 (3.2) | 6 (0.7) |  |
| High | 0 (0) | 20 (2.2) |  |
| Glandular |  |  | 0.0641 |
| No | 21 (67.7) | 662 (72) |  |
| Any | 10 (32.3) | 257 (28) |  |
| Low | 6 (19.4) | 216 (23.5) |  |
| Moderate | 4 (12.9) | 41 (4.5) |  |
| Articular |  |  | 0.00743 |
| No | 22 (71) | 548 (59.8) |  |
| Any | 9 (29) | 369 (40.2) |  |
| Low | 8 (25.8) | 282 (30.8) |  |
| Moderate | 0 (0) | 77 (8.4) |  |
| High | 1 (3.2) | 10 (1.1) |  |
| Cutaneous |  |  | 1.49e-05 |
| No | 22 (71) | 842 (91.4) |  |
| Any | 9 (29) | 79 (8.6) |  |
| Low | 5 (16.1) | 5 (0.5) |  |
| Moderate | 3 (9.7) | 68 (7.4) |  |
| High | 1 (3.2) | 6 (0.7) |  |
| Pulmonary |  |  | 0.0289 |
| No | 31 (100) | 865 (93.9) |  |
| Any | 0 (0) | 56 (6.1) |  |
| Low | 0 (0) | 22 (2.4) |  |
| Moderate | 0 (0) | 30 (3.3) |  |
| High | 0 (0) | 4 (0.4) |  |
| Renal |  |  | 0.621 |
| No | 30 (96.8) | 904 (98.2) |  |
| Any | 1 (3.2) | 17 (1.8) |  |
| Low | 1 (3.2) | 8 (0.9) |  |
| Moderate | 0 (0) | 3 (0.3) |  |
| High | 0 (0) | 6 (0.7) |  |
| Muscular |  |  | 1 |
| No | 31 (100) | 916 (99.5) |  |
| Any | 0 (0) | 5 (0.5) |  |
| Low | 0 (0) | 1 (0.1) |  |
| Moderate | 0 (0) | 2 (0.2) |  |
| High | 0 (0) | 2 (0.2) |  |
| Peripheral nervous system |  |  | 0.335 |
| No | 29 (93.5) | 874 (94.7) |  |
| Any | 2 (6.5) | 47 (5.1) |  |
| Low | 1 (3.2) | 4 (0.4) |  |
| Moderate | 1 (3.2) | 34 (3.7) |  |
| High | 0 (0) | 9 (1) |  |
| Central nervous system |  |  | 0.0782 |
| No | 28 (90.3) | 904 (98.2) |  |
| Any | 3 (9.7) | 17 (1.8) |  |
| Moderate | 2 (6.5) | 8 (0.9) |  |
| High | 1 (3.2) | 9 (1) |  |

* Fisher’s exact test for contingency tables

Abbreviations: ESSDAI, EULAR Sjögren Syndrome Disease Activity Index

Supplementary Table 4:

Percentage of patients in each ESSDAI score categories and level of ESSDAI activity: Comparison with adult patients reported previously [3]

|  | | Patients in each category (%) | |  |
| --- | --- | --- | --- | --- |
|  | | Children  n = 31 | Adults [3]  n = 1045 | *P*-value* |
| Level of the cumulative ESSDAI score | | | | |
| Low | 0 | 1 (3.2) | 208 (19.9) | 2.01e-06 |
|  | 1-4 | 6 (19.4) | 377 (36.1) |  |
| Medium | 5-13 | 15 (48.4) | 338 (32.3) |  |
| High | ≥14 | 9 (29.0) | 122 (11.7) |  |
| Maximum level of activity achieved in any of the 12 ESSDAI domains | | | | |
| None | | 1 (3.2) | 208 (19.9) | 1.49e-05 |
| Low | | 8 (25.8) | 401 (38.4) |  |
| Moderate | | 19 (61.3) | 334 (32.0) |  |
| High | | 3 (9.7) | 102 (9.7) |  |

* Fisher exact test for contingency tables

Abbreviations: ESSDAI, EULAR Sjögren Syndrome Disease Activity Index
