# Supplementary material for: Utility of the EULAR Sjögren syndrome disease activity index in Japanese children: a retrospective multicenter cohort study
Source: Pediatr Rheumatol Online J. 2020 Sep 17;18:73. doi: 10.1186/s12969-020-00458-1 (PMC7499954; doi:10.1186/s12969-020-00458-1)
Supplement: Supplementary file 3 — Additional file 3: Supplementary Table. The type of immunosuppressants are listed below and shown in the manuscript (Page 11, line 1-4). [file 12969_2020_458_MOESM3_ESM.docx]

Supplementary Table: The type of immunosuppressants are listed below and shown in the manuscript (Page 11, line 1-4).

| No. of Patient | Domains with more than moderate activity score | Levels of maximum ESSDAI activity | Glucocorticoid dosages | IM |
| --- | --- | --- | --- | --- |
| 1 | Central nervous system, Constitutional, Glandular, Biological | High | High  (PSL ≥ 0.5 mg/kg/day) | Tacrolimus |
| 10 | Constitutional, Biological | Moderate | Medium  (PSL ≥0.2, < 0.5 mg/kg/day) | Mizoribine |
| 14 | Glandular, Biological | Moderate | High  (PSL ≥ 0.5 mg/kg/day) | Mizoribine |
| 16 | Constitutional, Articular | High | High  (PSL ≥ 0.5 mg/kg/day) | Methotrexate |
| 18 | Cutaneous, Peripheral nervous system | Moderate | Medium  (PSL ≥0.2, < 0.5 mg/kg/day) | Methotrexate |
| 31 | Glandular, Hematological, Biological | High | Medium  (PSL ≥0.2, < 0.5 mg/kg/day) | Mycophenolate mofetil |
